# Supplementary figures and images for: Serum Amyloid P inhibits single stranded RNA-induced lung inflammation, lung damage, and cytokine storm in mice
Source: PLoS One. 2021 Jan 22;16(1):e0245924. doi: 10.1371/journal.pone.0245924 (PMC7822324; doi:10.1371/journal.pone.0245924)

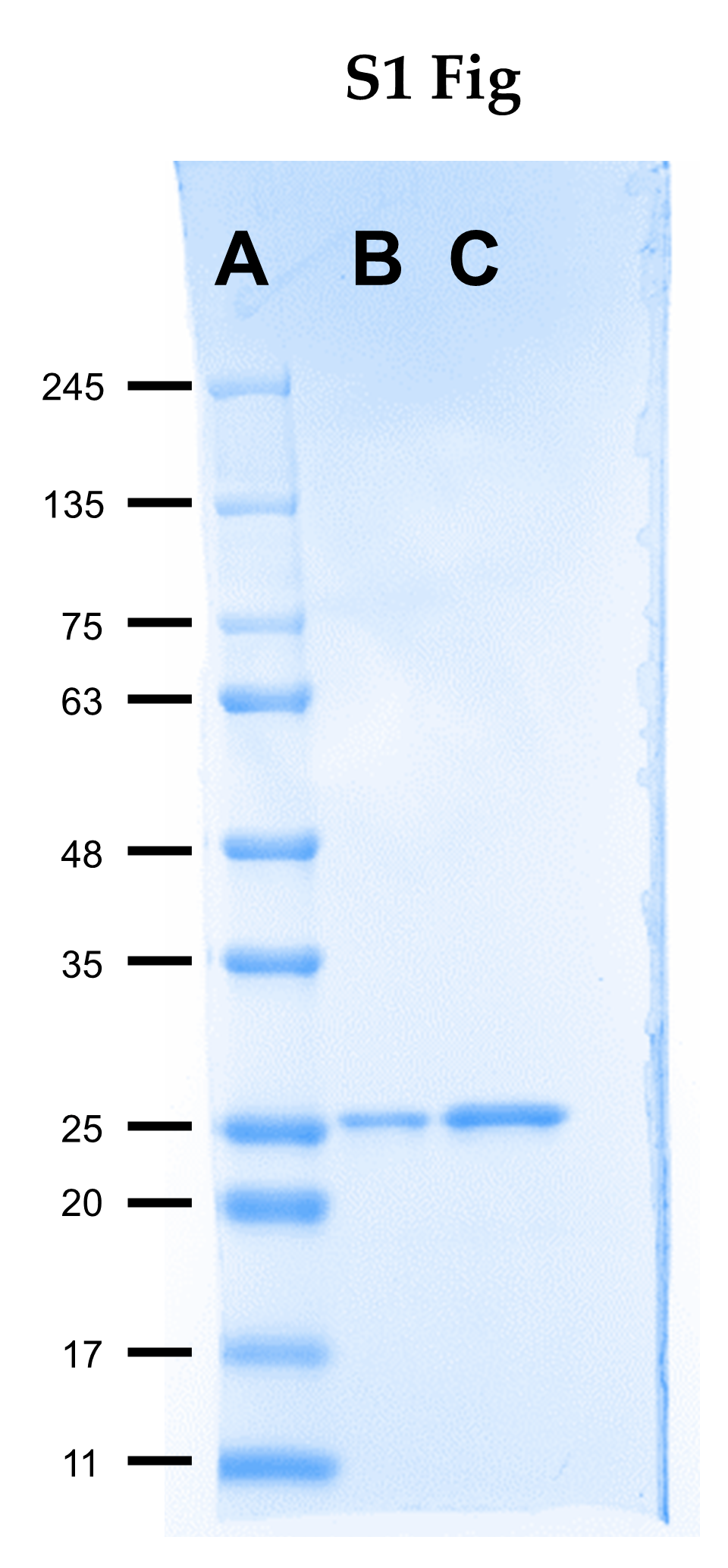

Supplement: S1 Fig — As described in the materials and methods section, the purified human SAP showed a single band at the expected molecular mass of ~26 kiloDaltons (kDa). Lane A is protein molecular mass marker with masses indicted in kDa. Lanes B and C are purified recombinant human SAP. (TIF) [file pone.0245924.s001.tif]

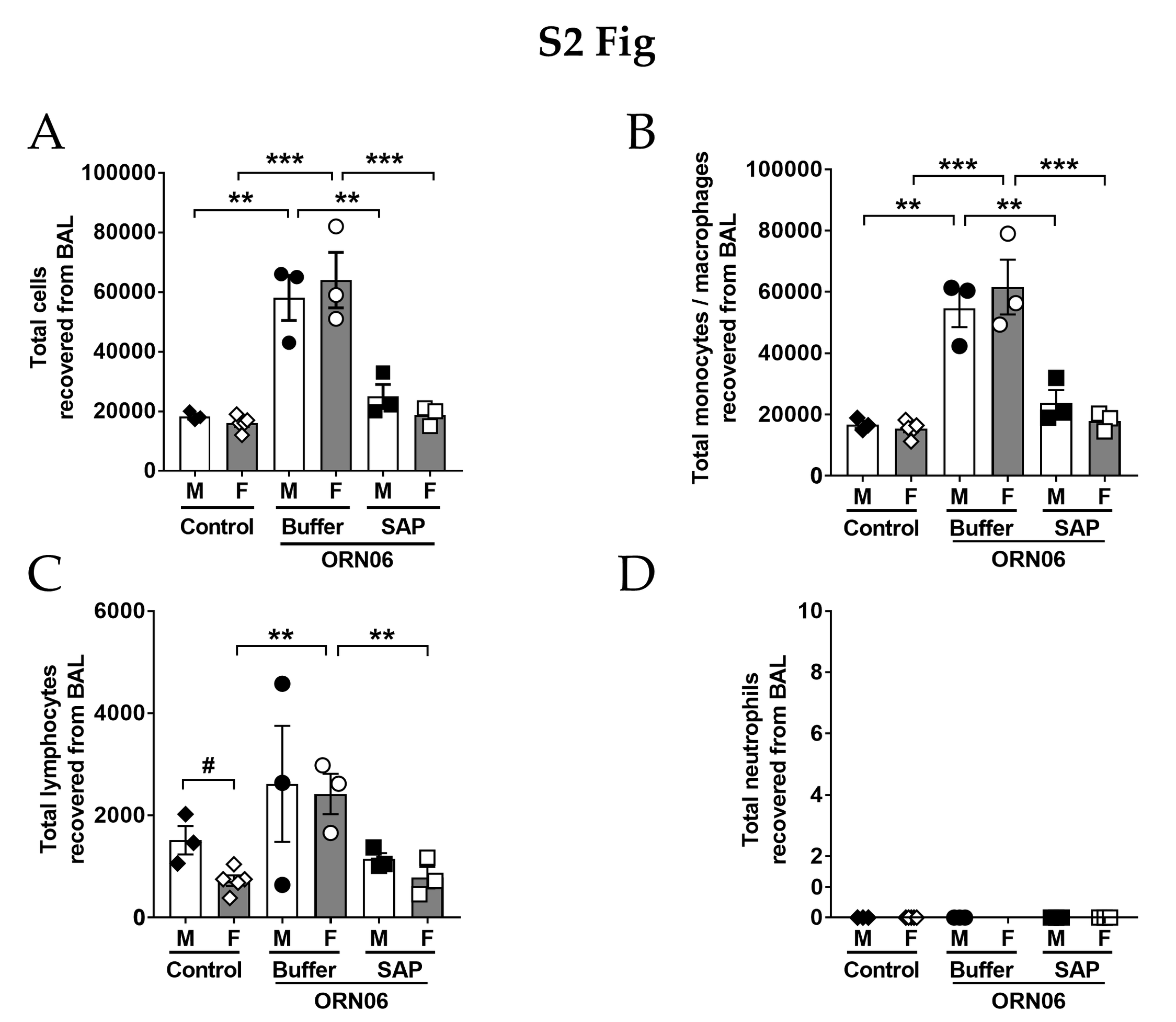

Supplement: S2 Fig — (A) The data from Fig 1A–1D were separated for male (M) and female (F) mice from each group for (A) total cells, (B) monocytes/ macrophages, (C) lymphocytes, and (D) neutrophils. Values are mean ± SEM. For male mice n = 3 in each group and for female mice n = 3 except for female mice control group, where n = 5. ** p < 0.01 and *** p < 0.001 (1-way ANOVA, Bonferroni’s test). # p < 0.05 (t-test). (TIF) [file pone.0245924.s002.tif]

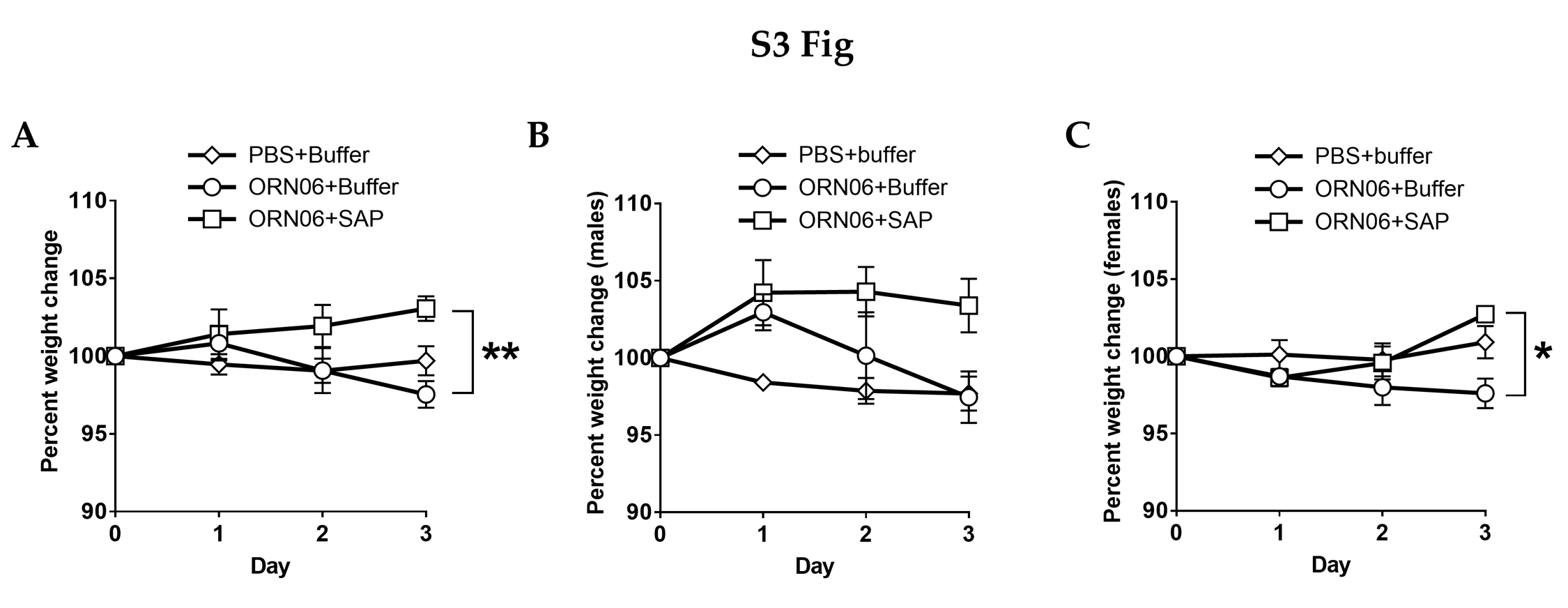

Supplement: S3 Fig — Percent change in body weight for (A) both males and females, (B) males, and (C) females after the indicated treatments. Values are mean ± SEM, n = 6 (3 males and 3 females) except for PBS-aspirated and then PBS-treated, where n = 8 (3 males and 5 females). * p < 0.05 and ** p < 0.01 (1-way ANOVA, Bonferroni’s test). (TIF) [file pone.0245924.s003.tif]

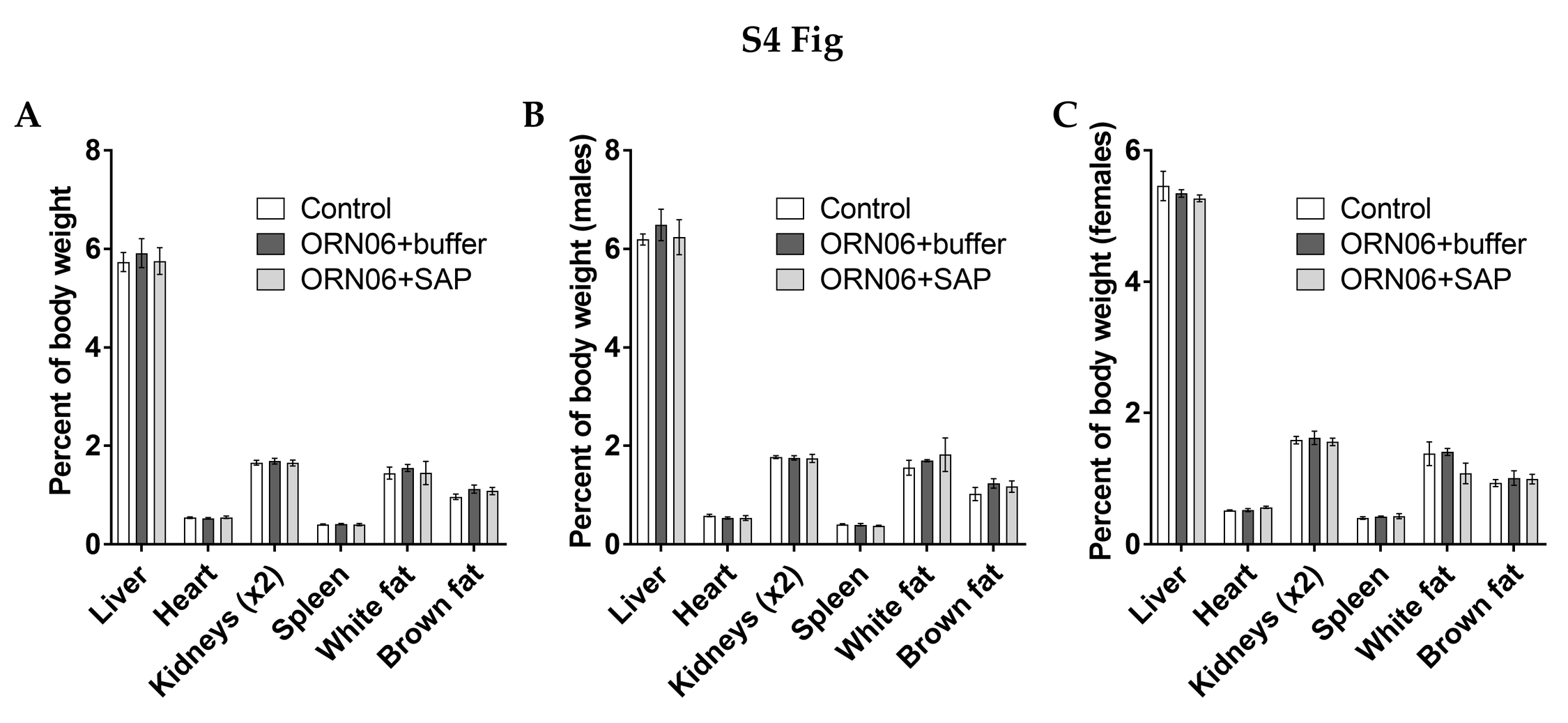

Supplement: S4 Fig — Organ weights as percent of total body weight at day 3 of (A) both male and female mice, (B) males, and (C) females after the indicated treatments. Values are mean ± SEM, n = 6 (3 males and 3 females) except for control, where n = 8 (3 males and 5 females). (TIF) [file pone.0245924.s004.tif]

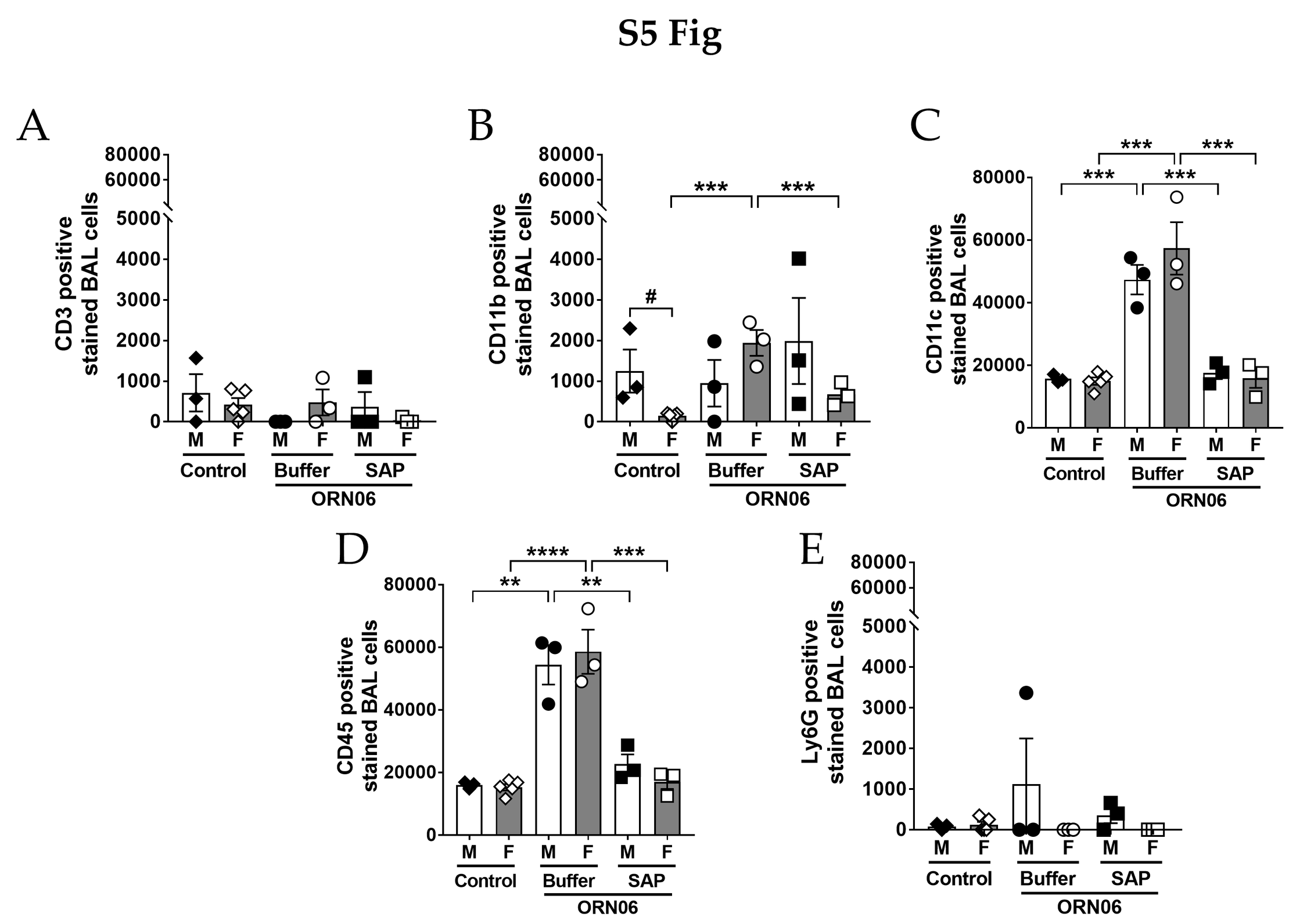

Supplement: S5 Fig — The data from Fig 2 was separated for (A) CD3 positively stained cells, (B) CD11b positively stained cells, (C) CD11c positively stained cells, (D) CD45 positively stained cells, and (E) Ly6G positively stained cells from male (M) and female (F) mice from each group. Values are mean ± SEM. For male mice n = 3 and for female mice n = 3 except for female mice control group, where n = 5. ** p < 0.01, *** p < 0.001, and **** p < 0.0001 (1-way ANOVA, Bonferroni’s test). # p < 0.05 (t-test). (TIF) [file pone.0245924.s005.tif]

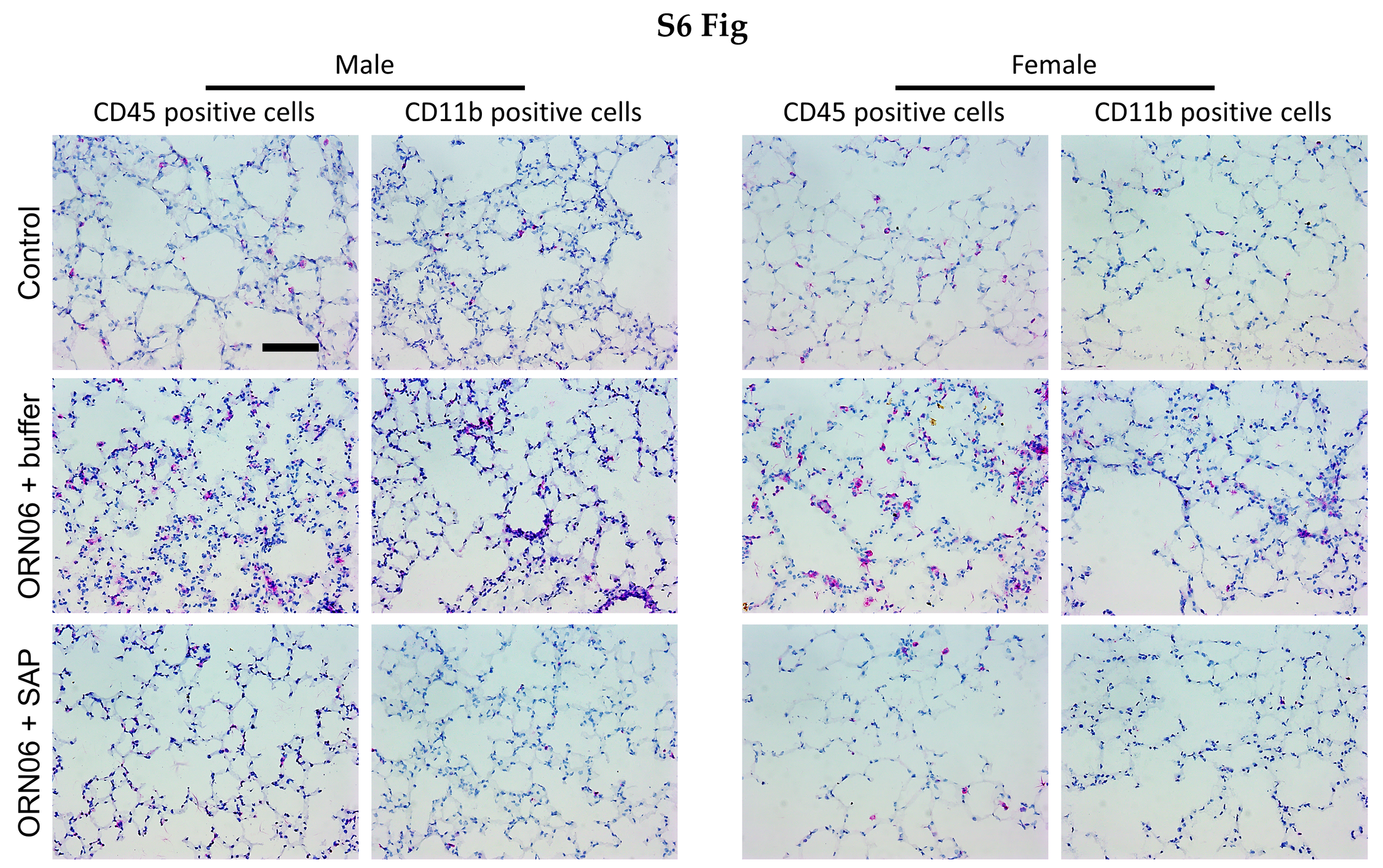

Supplement: S6 Fig — Representative images of male and female mouse lung cryosections stained with antibodies against CD45 and CD11b. Red is staining, blue is counter stain. Bar is 100 μm. Images are representative of n = 3 in each male and female group, except for female mouse control group, where n = 4. (TIF) [file pone.0245924.s006.tif]

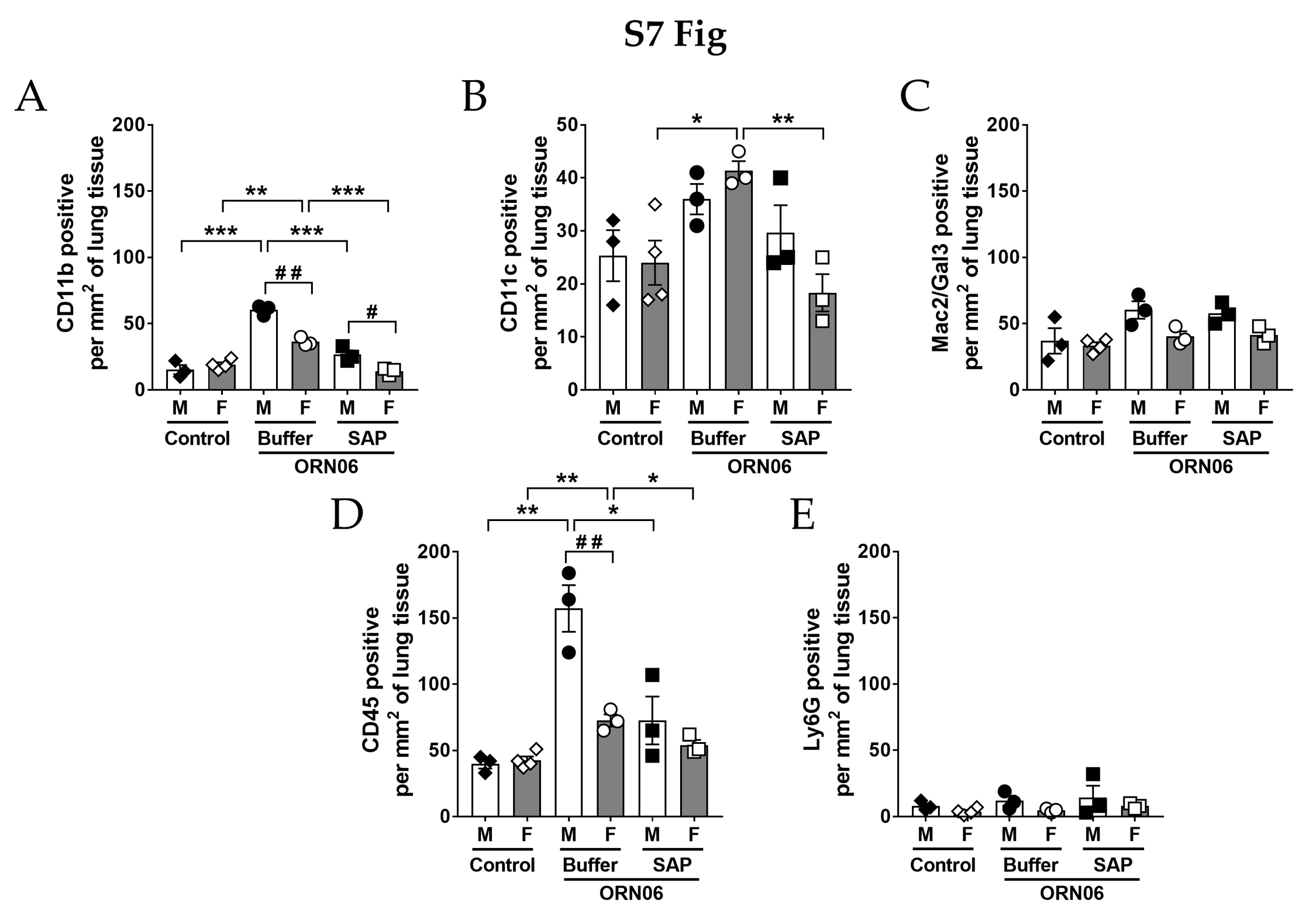

Supplement: S7 Fig — The data from Fig 3 was separated for (A) CD11b positively stained cells, (B) CD11c positively stained cells, (C) Mac2/Gal3 positively stained cells, (D) CD45 positively stained cells, and (E) Ly6G positively stained cells from male (M) and female (F) mice from each group. Values are mean ± SEM. For male mice n = 3 and for female mice n = 3 except for female mice control group, where n = 4. * p < 0.05, ** p < 0.01, and *** p < 0.001 (1-way ANOVA, Dunnett’s test). # p < 0.05 and ## p < 0.01 (t-test). (TIF) [file pone.0245924.s007.tif]

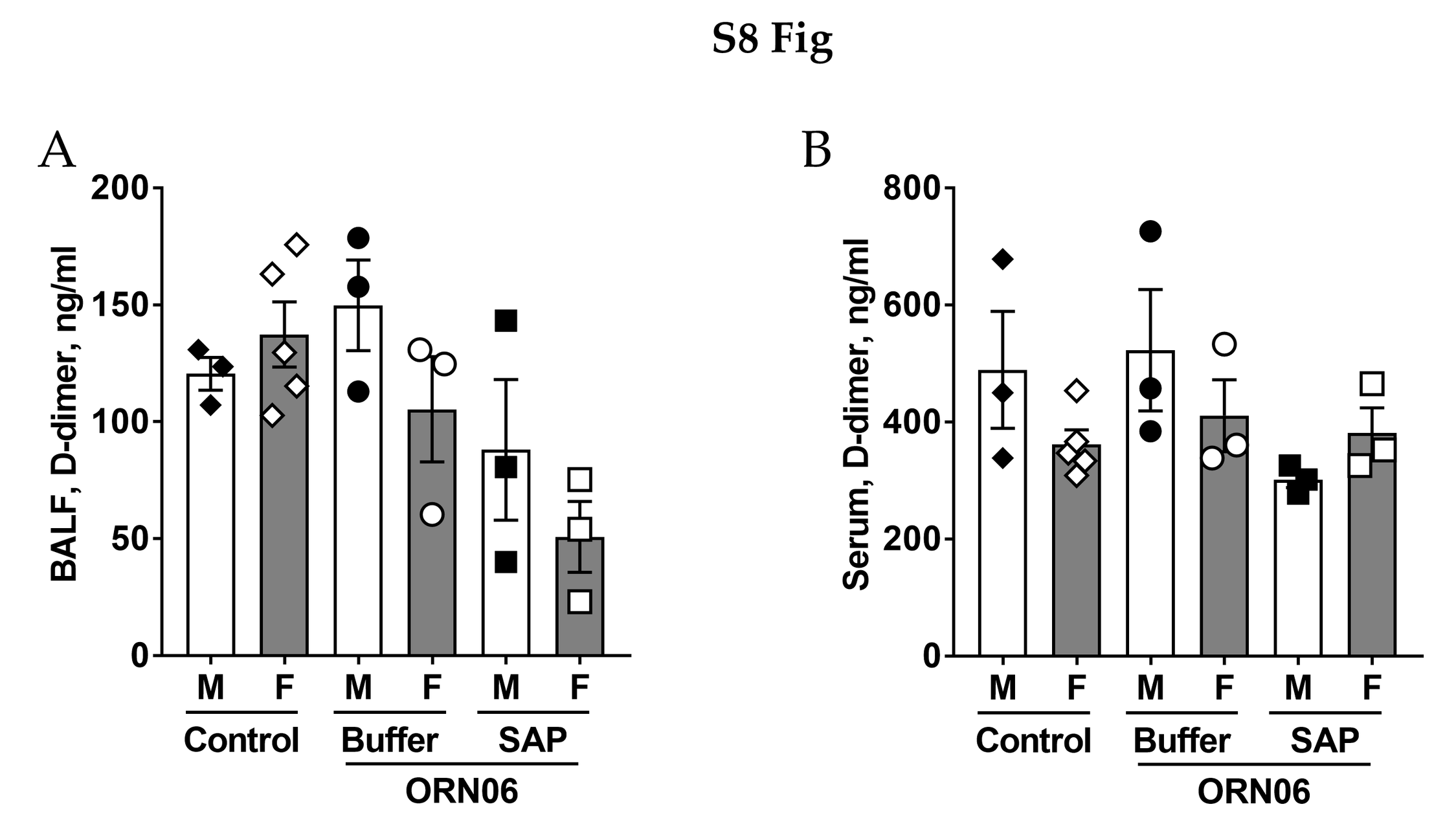

Supplement: S8 Fig — The data from Fig 4B and 4C were separated for (A) BALF and (B) Serum D-dimers levels from male (M) and female (F) mice from each group. (TIF) [file pone.0245924.s008.tif]

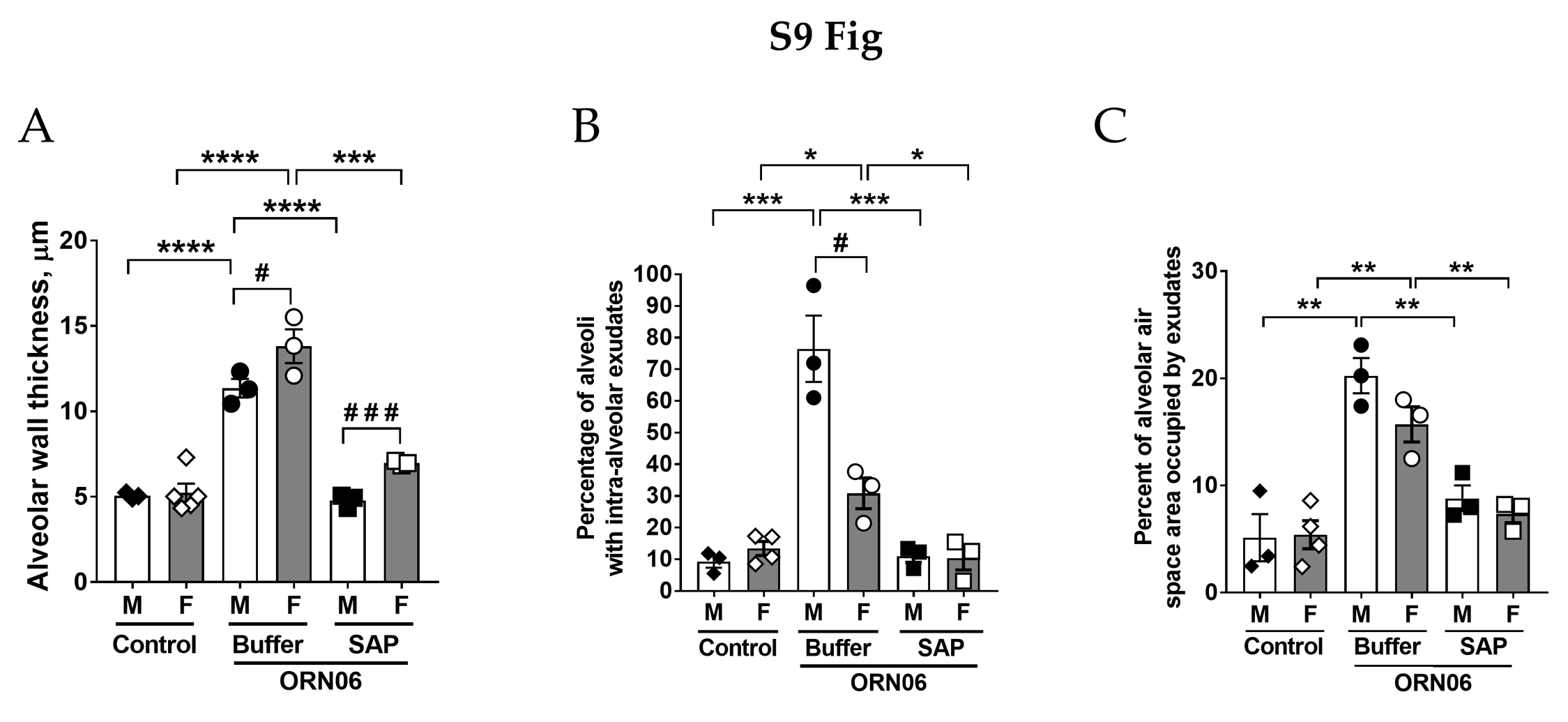

Supplement: S9 Fig — The data from Fig 5D, 5E and 5F were separated for (A) alveolar wall thickness, (B) percent of alveoli with intra-alveolar exudates, and (C) percent of alveolar airspace area occupied by exudates from male (M) and female (F) mice from each group. Values are mean ± SEM. For male mice n = 3 in each group and for female mice n = 3 except for female mice control group in (A), where n = 5 and in (B–C), where n = 4. * p < 0.05, ** p < 0.01, *** p < 0.001, and **** p < 0.0001 (1-way ANOVA, Bonferroni’s test). # p < 0.05, ### p < .001 (t-test). (TIF) [file pone.0245924.s009.tif]
